# Maternal immunization increases nestling energy expenditure, immune function, and fledging success in a passerine bird

**DOI:** 10.1242/bio.028803

**Published:** 2018-04-15

**Authors:** Gary Burness, Deanna Moher, Noah Ben-Ezra, Ryan J. Kelly, Dennis Hasselquist, Eunice H. Chin

**Affiliations:** 1 Department of Biology, Trent University, Peterborough, Ontario K9L 0G2, Canada; 2 Department of Animal Ecology, Lund University, S-223 63 Lund, Sweden; 3 Environmental and Life Science Graduate Program, Trent University, Peterborough, Ontario K9L 0G2, Canada

**Keywords:** Energetics, Growth, Lipopolysaccharide, Fitness, Maternal antibody transmission, Maternal effects, Mismatch hypothesis

## Abstract

Female birds transfer maternally derived antibodies (matAb) to their nestlings, via the egg yolk. These antibodies are thought to provide passive protection, and allow nestlings to avoid the costs associated with mounting an innate immune response. To test whether there is an energetic benefit to nestlings from receiving matAb, we challenged adult female tree swallows (*Tachycineta bicolor*) prior to clutch initiation with either lipopolysaccharide (LPS) or saline (Control). Following hatching, one half of each female's nestlings were immunized on day 8 post-hatch with LPS or saline, and the 4-h post-immunization nestling metabolic rate (MR) was measured. There was no difference in either LPS-reactive antibodies or total Ig levels between offspring of immunized and non-immunized mothers on day 6 or 14 post-hatch, possibly reflecting a relatively short half-life of matAbs in altricial birds. Additionally, we found no evidence that nestlings from LPS-immunized mothers could avoid the growth suppression that may result from activation of an inflammatory response. Unexpectedly, we found that control nestlings from LPS mothers had higher resting MR than control nestlings of control mothers. We attribute the increased MR to the costs associated with a general non-specific enhancement of immune function in nestlings from LPS-immunized mothers. Consistent with enhanced immune function, nestlings of immunized mothers had a more robust inflammatory response to phytohaemagglutinin and higher fledging success. Our results suggest that maternal antigen exposure pre-laying can result in increased fitness for both mothers and offspring, depending on food availability.

## Abstract (precis)

**Summary:** Exposure of female birds to a simulated pathogen prior to egg laying increases the metabolic rate, immune function, and fledging success of her nestlings.

## INTRODUCTION

The prenatal environment is increasingly recognized as an important source of phenotypic variation (Benowitz-Fredericks et al., 2015). Maternal effects, the non-genomic influences a female has on her offspring, represent a key aspect of this environment (Mousseau and Fox, 1998). During embryonic development, offspring can be exposed to varied maternally derived compounds, including hormones (Tschirren et al., 2014), nutrients (Moreno et al., 2008; Nelson et al., 2010), and antibodies (Hasselquist and Nilsson, 2009). Via such compounds, mothers have been hypothesized to transfer information about prevailing environmental conditions to offspring (Boulinier and Staszewski, 2008). Because mothers and offspring often share the same environment, this transgenerational effect may be an important source of adaptive phenotypic plasticity (Agrawal et al., 1999).

There has been increasing interest in the role that maternally derived antibodies (matAbs) have on offspring immune system development and subsequent offspring fitness (reviewed in Hasselquist et al., 2012). In birds, although nestlings start to produce specific antibodies during the first few weeks of life (Hasselquist and Nilsson, 2009), the adaptive immune system fully matures over much longer time periods (Killpack and Karasov, 2012). Thus, during the most vulnerable 1- to 2-week period of early life, offspring are thought to rely on the presence of matAbs for passive protection, transferred to them via the egg yolk (Hasselquist and Nilsson, 2009). The level and specificity of matAbs transferred reflects the prior exposure of the mother to antigens; if the female has not been exposed to a given pathogen, she is unable to confer passive antibody protection to her offspring (Lemke et al., 2003). Although nestlings may rapidly catabolize matAbs (King et al., 2010), the duration of antibody persistence in nestling plasma reflects the levels in maternal circulation and the levels transferred to the egg (Grindstaff, 2010). Maternal antibodies can continue to be transferred to offspring many months after mothers have been exposed (Reid et al., 2006), and even during successive breeding bouts for many years after the female's initial exposure to a pathogen (Ramos et al., 2014). In birds, matAb transfer is thought to mainly involve IgG (Grindstaff et al., 2003).

In the absence of antigen-specific antibodies, nestlings that encounter a pathogen must rely on activation of the non-specific innate immune system, and the accompanying inflammatory response (Grindstaff, 2008). The response is rapid, but is considered costly due to factors such as the generation of a fever, anorexia, and suppression of growth (Klasing and Leshchinsky, 1999). Experimentally, the presence of matAbs has been shown to reduce the inflammatory response, allowing individuals to offset growth suppression (Klasing and Leshchinsky, 1999; Grindstaff, 2008). Given the energetic costs of mounting an inflammatory response (reviewed in Hasselquist and Nilsson, 2012), the passive protection afforded by matAbs may allow offspring to reduce energy expenditure, and reallocate energy toward growth and tissue maturation (Grindstaff, 2008). However, whether matAbs can ameliorate the energetic costs of mounting an immune response has, to our knowledge, not been tested.

MatAbs transferred to offspring not only provide passive protection, but can also prime the offspring's immune systems to deal with subsequent infections (Anderson, 1995; Lemke et al., 2004, 2009). This priming can be specific, resulting in increased production of specific antibodies when individuals subsequently encounter the same antigen (Lemke et al., 2003; Reid et al., 2006; Grindstaff, 2008; Broggi et al., 2016) or non-specific, where there is a general priming of the individual's humoral immune system (Grindstaff et al., 2006). Additionally, matAbs have also been shown to block antibody production in offspring, leading to a subsequent suppression of humoral immunity (Carlier and Truyens, 1995; Siegrist, 2003; Staszewski et al., 2007; Merrill and Grindstaff, 2014). The differing responses likely reflect such factors as the amount of matAb transferred to the offspring and their persistence, dose of antigen, and species-specific life-history strategies (Siegrist, 2003; Elazab et al., 2009; Hasselquist and Nilsson, 2009; Niewiesk, 2014).

In the current study, we examined the effect of maternal immunization on the subsequent energy expenditure and immunological development of nestling tree swallows, a declining aerial insectivore (Nebel et al., 2010). We challenged pre-laying females and 8-day-old nestlings with lipopolysaccharide (LPS) or saline (Control). We then measured nestling growth rate, total and antigen-reactive antibody levels, energy expenditure, and subsequent inflammatory response near fledging. LPS is a major component of the outer membrane of Gram-negative bacteria, and immunization with LPS induces an acute phase response with a 10-15% increase in metabolic rate (Burness et al., 2010). Because LPS is a type-1 thymus-independent antigen (TI-1), it does not require T cell help to produce antibodies (Murphy and Weaver, 2016). As a result, LPS can induce a relatively rapid antibody response, within 5 days of immunization (Sköld-Chiriac et al., 2014). Importantly, at least some type of LPS-reactive antibodies can be transmitted to offspring via the egg yolk (Sunwoo et al., 1996), and these antibodies should mainly be of the IgG type (Grindstaff et al., 2003); IgM antibodies are absent in the yolk (Rose et al., 1974) and therefore cannot be taken up by the embryo/neonate.

We hypothesized that maternal immunization would influence offspring immune function and energy utilization. We predicted that nestlings of immunized mothers that were challenged with the same antigen as their mother would: (1) avoid the growth-suppressive effects of generating an innate immune response, and grow faster than nestlings of non-immunized mothers; (2) avoid the metabolic cost of an innate immune response, and thus expend less energy while generating an inflammatory response than nestlings from non-immunized mothers; (3) generate a stronger response to a subsequent immune challenge (phytohaemagglutinin, PHA) than nestlings from non-immunized mothers, due to non-specific priming of immunity.

## RESULTS

### No effect of maternal immunization on timing of clutch initiation, clutch size or hatching success

We injected 61 females with LPS or saline (2009: LPS, *n*=19; Saline, *n*=11; 2010: LPS, *n*=17; Saline, *n*=14). Of these, 26 females were later located in nest boxes and laid eggs at least 5 days post-immunization (2009: LPS, *n*=7; Saline, *n*=7; 2010: LPS, *n*=6; Saline, *n*=6). Females initiated egg laying (mean±s.e.), 9.62±0.60 days post-capture (range 5-17 days), and this did not differ between treatments [LPS females, 10.0±0.90 days, *n*=13; Control females, 9.23±0.82 days, *n*=13; t=0.633, *P*=0.532, degrees of freedom (d.f.)=23.79]. Only two females initiated egg laying 5 days post-immunization (one from each maternal treatment); all other females initiated clutches at least 7 days post-immunization. The average clutch size was 5.54±0.16 eggs (range 4-7 eggs), and did not differ between treatments (LPS-females, 5.38±0.24 eggs; Control females, 5.69±0.21 eggs; t=0.965, *P*=0.344, d.f.=23.50). Clutch size and the number of days to initiate egg laying also did not differ between years of study (Clutch size: t=1.19, *P*=0.249, d.f.=21.31; Number of days: t=0.612, *P*=0.548, d.f.=20.59). There was no effect of maternal immunization on hatching success [LPS mothers: 84.6±6.6%, *n*=13; Control mothers: 90.1±3.5%, *n*=13; general linear mixed model (GLMM), t=0.201, *P*=0.842, d.f.=24]. Maternal mass did not differ between treatments (*F*_1,23_=0.544, *P*=0.468), between pre-laying and incubation (Breeding period: *F*_1,24_=1.893, *P*=0.182), and immunization had no effect on maternal mass change between immunization and mid-incubation (Maternal treatment×Breeding period, *F*_1,24_=0.039, *P*=0.846). Maternal mass was higher in 2009 than 2010 (Year, *F*_1,23_=5.171, *P*=0.033).

### No effect of maternal immunization on maternal antibody levels

On initial capture, LPS-immunized and control females did not differ in background levels of LPS-reactive antibodies (*F*_1,21_=0.361, *P*=0.554; Year, *F*_1,21_=29.375, *P*<0.001). When individuals were re-captured during mid-incubation, the level of LPS-reactive antibodies had not increased (Breeding period: *F*_1,23.77_=0.766, *P*=0.390). LPS-immunized and control females did not differ in their levels of LPS-reactive antibodies (Maternal treatment: *F*_1,21.92_=0.826, *P*=0.373), although females had higher levels in 2010 than in 2009 (*F*_1,21.79_=15.330, *P*<0.001). On average (mean±1 s.e.), 20.7±0.63 days (range 15-28 days) elapsed between maternal immunization pre-laying and subsequent re-capture and blood sampling during mid-incubation. Levels of LPS-reactive antibodies were not predicted by the number of days that had elapsed between maternal immunization and when a female was recaptured (*F*_1,21.41_=1.526, *P*=0.230).

Total Ig concentrations did not differ between LPS-immunized and control females upon initial capture (*F*_1,22_=0.007, *P*=0.936). In contrast to LPS-reactive antibodies, the concentrations of total Ig increased between pre-laying and incubation in most individuals (19 of 24 individuals for which we had both blood samples; Breeding period: *F*_1,21.99_=15.051, *P*<0.001). This increase did not differ between females immunized with LPS and control females (Breeding period×Maternal treatment: *F*_1,21.99_=0.126, *P*=0.726). On average, total Ig levels did not differ between LPS-immunized and control females (Maternal treatment: *F*_1,22.77_=0.012, *P*=0.914). Total Ig levels by mid-incubation were not predicted by the number of elapsed days between maternal immunization and subsequent blood sampling (*F*_1,22.62_=0.481, *P*=0.495).

### No effect of maternal immunization on antibody production in nestlings

At 6 days of age, nestlings whose mothers had been immunized with LPS had similar levels of LPS-reactive antibody titers to nestlings from control mothers (Maternal treatment: *P*=0.318, Year: *P*<0.001; Table 1); with levels higher in 2009 than in 2010. By postnatal day 14, there had been a significant increase in LPS-reactive antibodies in 97% of nestlings (104 of 107 individuals; Paired *t*-test, t=17.792, d.f.=106, *P*<0.001), and within nestlings there was a positive correlation between levels on days 6 and 14 (β=0.61, *F*_1,102.4_=58.885, *P*<0.001). However, LPS-reactive antibody levels at postnatal day 14 were not affected by maternal treatment, nestling treatment, or their interaction (all *P*>0.30; Table 1). The number of elapsed days between maternal immunization and the start of egg laying did not affect LPS-reactive antibody titers at either day 6 or day 14 (Day 6: *F*_1,17.85_=0.108, *P*=0.747; Day 14: *F*_1,18.82_=2.016, *P*=0.172).

**Table 1.**
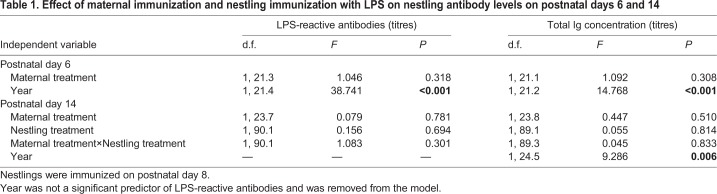
**Effect of maternal immunization and nestling immunization with LPS on nestling antibody levels on postnatal days 6 and 14**

Total Ig levels in 6-day-old nestlings did not differ between nestlings whose mothers were immunized with LPS and nestlings of control mothers (Maternal treatment: *P*=0.308), but levels were higher in 2010 than 2009 (Year: *P*<0.001; Table 1). By day 14, total Ig levels had increased in most individuals (104 of 107 individuals, Paired *t*-test, t=18.377, d.f.=106, *P*<0.001), and there was a positive relation between Ig levels on days 6 and 14 (β=0.42, *F*_1,74.61_=62.642, *P*<0.001). Total Ig levels on postnatal day 14 were unrelated to maternal treatment, nestling treatment, or their interaction (all *P*>0.50; Table 1), but levels were higher in 2010 than 2009 (*P*=0.006; Table 1). The number of elapsed days between maternal immunization and clutch initiation did not affect total Ig levels in nestlings at either day 6 or day 14 (Day 6: *F*_1,17.04_=1.970, *P*=0.178; Day 14: *F*_1,19.41_=0.523, *P*=0.478).

There was a significant positive relation between nestling total Ig levels and LPS-reactive antibodies at day 6 (β=0.44, *F*_1,111.1_=29.714, *P*<0.001; Year: *F*_1,31.7_=44.221, *P*<0.001) and day 14 post-hatch (β=0.20, *F*_1,103.8_=8.898, *P*=0.004; Year: *F*_1,35.26_=21.036, *P*<0.001). This suggests that LPS-reactive antibody levels reflect total Ig levels.

### Nestling growth rates were reduced for 24 h post-immunization

**Fig. 1.**
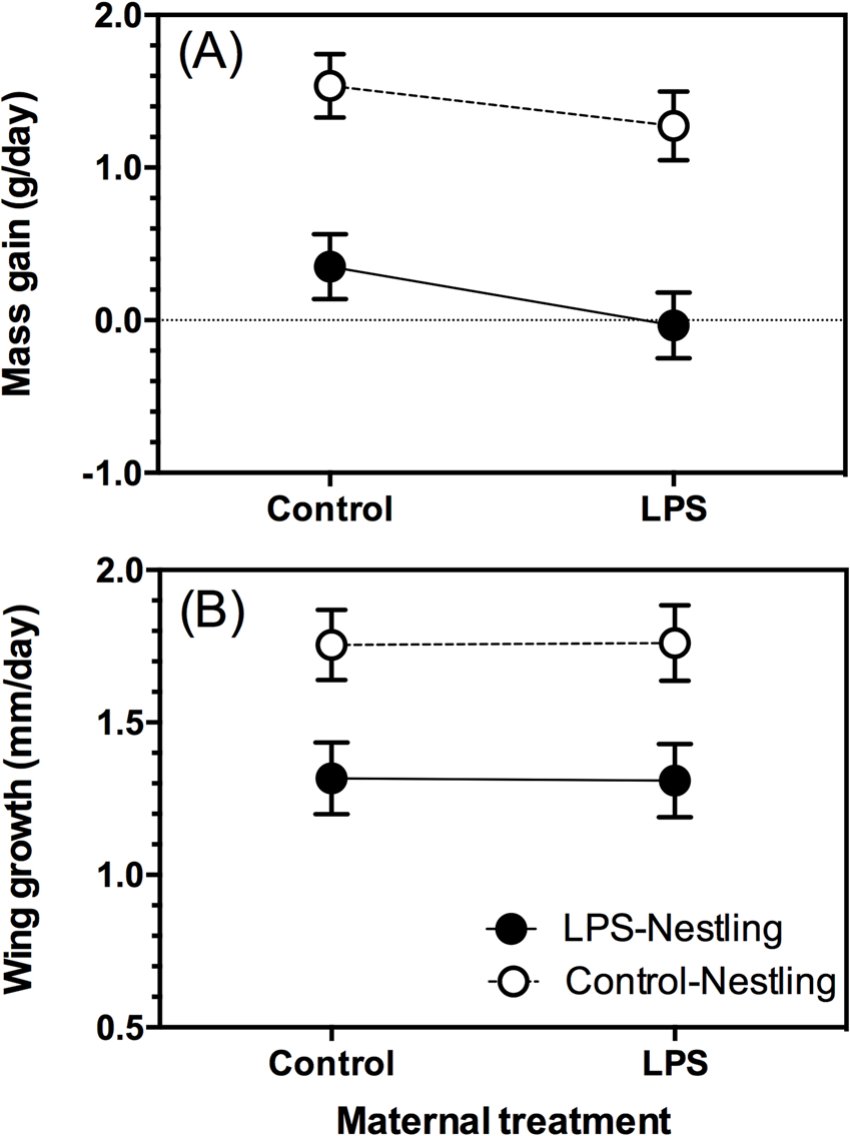
**Effect of maternal immunization with LPS on (A) mass gain and (B) wing growth of 8-day-old nestlings, during the 24 h following nestling LPS immunization.** Data were analyzed with GLMMs, with maternal identity as a random effect (see text for details). Maternal immunization had no significant effect on mass gain or wing growth (both *P*>0.05). Nestling immunization significantly depressed both measures of growth (both *P*<0.05). Maternal immunization was unable to ameliorate the negative effect of nestling immunization (indicated by a non-significant interaction between maternal and nestling immunization, *P*>0.05). Sample sizes (number of nests): LPS mothers, *n*=13; Control mothers, *n*=13. Sample sizes (number of nestlings): LPS mothers/LPS nestlings, *n*=29; LPS mothers/Control nestlings, *n*=26; Control mothers/LPS nestlings, *n*=31; Control mothers/Control nestlings, *n*=33. Data are presented as least squares means±1 s.e.m.

We weighed and measured nestlings on day 8 prior to immunization, and, 24 h later, the difference was considered a measure of short-term growth. Maternal immunization had no effect on nestling mass gain or wing growth following nestling immunization (mass gain, *F*_1,24.56_=1.654, *P*=0.210; wing growth, *F*_1,20.76_<0.001, *P*=0.999). Nestlings challenged with LPS gained less mass during this 24-h period than did control nestlings (nestling treatment: *F*_1,94.43_=52.274, *P*<0.001; Covariate, pre-immunization body mass: β=−0.15, *F*_1,96.06_=14.212, *P*<0.001; Fig. 1A), and their wings grew more slowly (nestling treatment: *F*_1,89.31_=27.612, *P*<0.001; Covariate, pre-immunization wing length: β=−0.09, *F*_1,106.3_=13.549, *P*<0.001; Fig. 1B). Maternal immunization with LPS did not offset the growth suppressive effects of nestling LPS-immunization (Maternal treatment×Nestling treatment: Mass gain, *F*_1,94.51_=0.120, *P*=0.730; Wing growth, *F*_1,89.41_=0.008, *P*=0.929; Fig. 1). Nestling mass gain differed between years of study (*F*_1,31.32_=6.219, *P*=0.018), although wing growth did not (*P*>0.05).

### Maternal immunization did not affect nestling growth rates throughout development

To explore the effect of maternal immunization on nestling growth rates, we divided growth into early (days 4 to 8) and late (days 9 to 14) phases. On postnatal day 4, offspring of LPS-immunized and control mothers did not differ in body mass (maternal treatment: *F*_1,14.02_=0.040, *P*=0.844) or wing length (Maternal treatment: *F*_1,14.26_=0.192, *P*=0.668), but differed between years and with brood size (each, *P*<0.01). Between postnatal days 4 and 8, nestlings increased in body mass and wing length (Age: *P*<0.001 for both; Table 2). Nestlings whose mothers had been immunized with LPS grew at a similar rate to nestlings from control mothers (as indicated by non-significant interactions between age and maternal treatment; Table 2). Nestlings were heavier and had longer wings in 2009 than in 2010 (Year, Table 2). Brood size at day 4 had a significant effect on nestling mass and wing length (Table 2). Females that delayed clutch initiation following immunization (i.e. let more days elapse) had lighter and smaller nestlings during early growth (Mass: β=–0.18±0.07, *P*=0.020; Wing length: β=−0.14±0.06, *P*=0.025; Table 2).

**Table 2.**
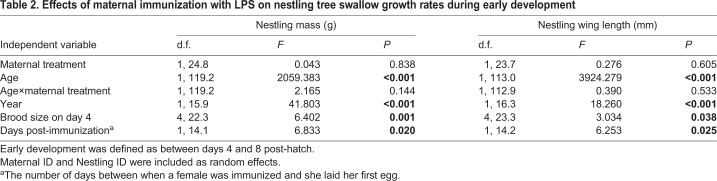
**Effects of maternal immunization with LPS on nestling tree swallow growth rates during early development**

Between postnatal days 9 and 14, nestling growth rates were not affected by maternal treatment or nestling treatment (Age×Maternal treatment, Age×Nestling treatment, respectively; Table 3). There was also no significant interaction between maternal treatment and nestling treatment for either nestling mass or wing length (Table 3). However, on average, nestlings of LPS immunized mothers had significantly longer wings than chicks of control mothers (Maternal treatment: LPS mothers, 28.19±0.61 mm, Control mothers: 26.89±0.62 mm, *P*=0.008; Table 3). Chicks were heavier (but did not have longer wings) in 2009 than 2010. Females that delayed clutch initiation following immunization (i.e. let more days elapse) had lighter and smaller nestlings during late growth (Mass: β=−0.26±0.11, *P*=0.031; Wing length: β=−0.29±0.06, *P*<0.001; Table 3).

**Table 3.**
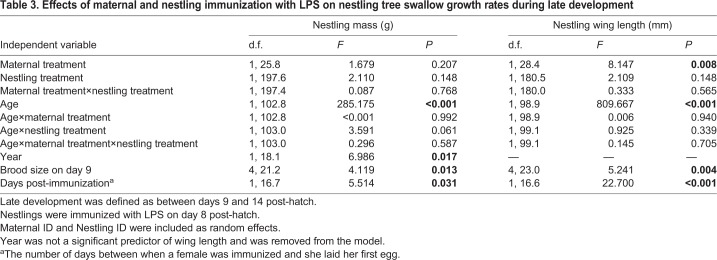
**Effects of maternal and nestling immunization with LPS on nestling tree swallow growth rates during late development**

At postnatal day 14, maternal treatment had no effect on nestling body mass or wing length (Mass: *F*_1,23.26_=0.151, *P*=0.702; Wing length, *F*_1,21.61_=0.180, *P*=0.676). Nestlings previously challenged with LPS on postnatal day 8 were lighter and smaller than control chicks, but this difference failed to attain statistical significance (Mass: *F*_1,86.66_=2.841, *P*=0.096; Wing length: *F*_1,86.22_=3.854, *P*=0.053). There was no significant interaction between maternal and nestling immunization, and nestling mass or wing length on postnatal day 14 (Mass: *F*_1,86.66_=0.864, *P*=0.355; Wing length: *F*_1,86.22_=2.523, *P*=0.116).

### Nestling metabolic rate was affected by the interaction between maternal and nestling immunization

**Fig. 2.**
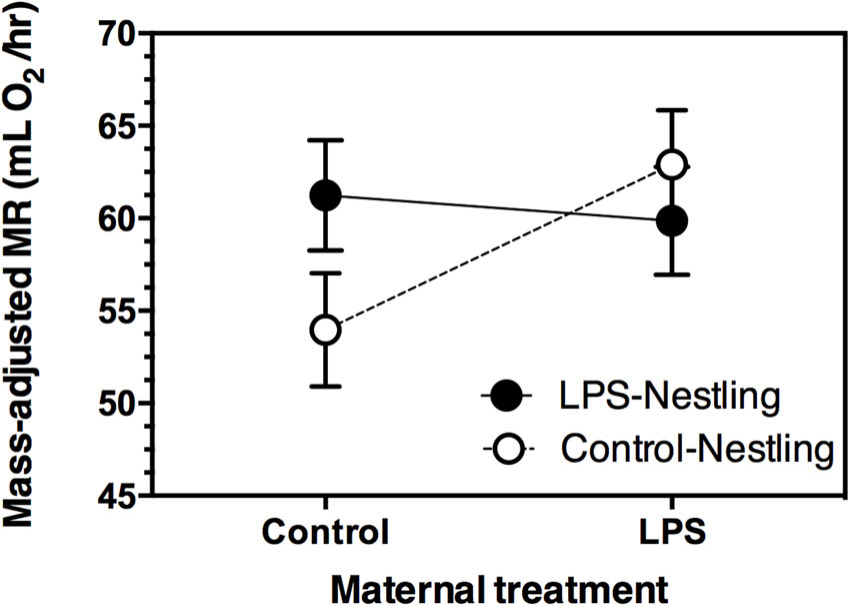
**Effect of maternal and nestling immunization with LPS on the mass-adjusted metabolic rate of 8-day-old tree swallow nestlings.** Data were analyzed with a GLMM, with maternal identity as a random effect, and nestling body mass as a covariate (see text for details). Maternal and nestling immunization did not independently affect mass-adjusted metabolic rate (both *P*>0.05), but there was a significant interaction between maternal and nestling immunization (*P*<0.05). Sample sizes (number of nests): LPS mothers, *n*=13; Control mothers *n*=13. Samples sizes (number of nestlings): LPS mothers/LPS nestlings, *n*=13; LPS mothers/Control nestlings, *n*=13; Control mothers/LPS nestlings, *n*=13; Control mothers/Control nestlings, *n*=12. Data are presented as least squares means±1 s.e.m.

Maternal treatment and nestling treatment did not independently affect nestling metabolic rate (Table 4). However, the metabolic response of nestlings to LPS immunization depended on whether their mothers had been immunized with LPS or not (Maternal treatment×Nestling treatment; Table 4, Fig. 2). Control nestlings from control mothers had lower metabolic rates than all other groups (planned post-hoc comparison, *F*_1,43.38_=5.129, *P*=0.029). Nestling metabolic rate increased with body mass (β=6.488, *P*<0.001), declined as the season progressed (hatch date: β=−1.164, *P*=0.011), and differed between years (being higher in 2009 than 2010; Table 4). Mass, hatch date, and year were retained in the final statistical model.

**Table 4.**
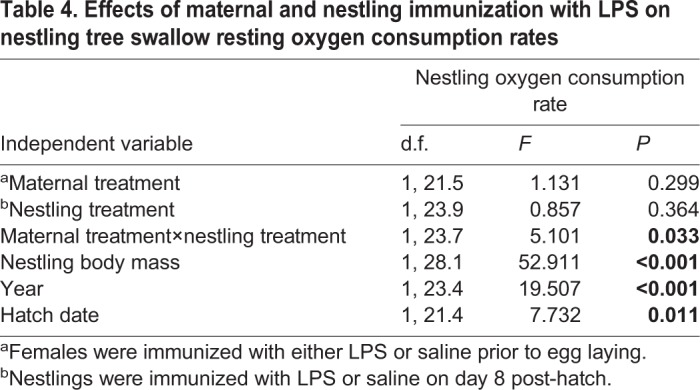
**Effects of maternal and nestling immunization with LPS on nestling tree swallow resting oxygen consumption rates**

### Maternal and nestling immunization affected nestling wing-web swelling response

**Fig. 3.**
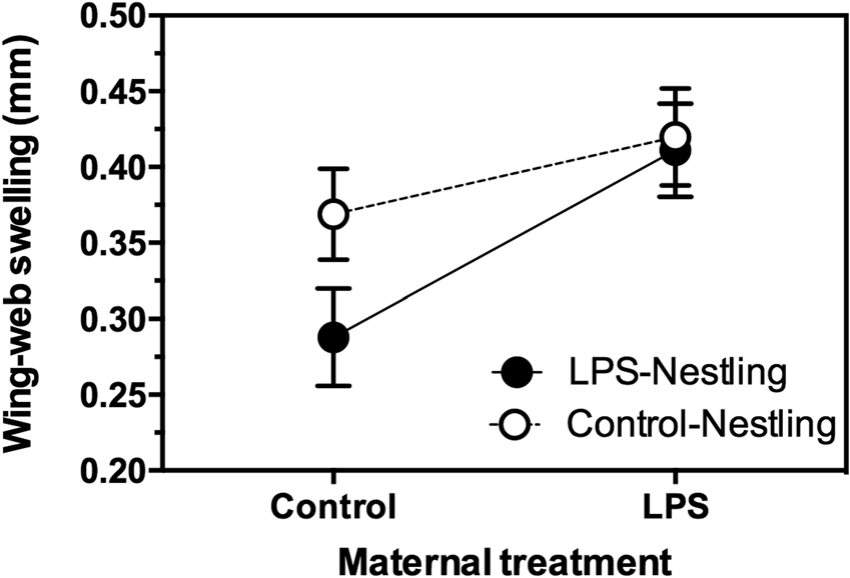
**Effect of maternal and nestling immunization with LPS on the subsequent wing-web swelling response to PHA of 14-day-old tree swallow nestlings.** Data were analyzed with a GLMM, with maternal identity as a random effect (see text for details). Maternal immunization with LPS significantly increased the nestlings' wing-web swelling response (*P*<0.05), while immunization of nestlings with LPS significantly depressed the nestlings' response to PHA (*P*<0.05). There was a trend for an interaction between maternal and nestling immunization with LPS, and nestlings' response to PHA (*P*=0.075). Sample sizes (number of nests): LPS mothers, *n*=13; Control mothers *n*=13. Sample sizes (number of nestlings): LPS mothers/LPS nestlings, *n*=29; LPS mothers/Control nestlings, *n*=26; Control mothers/LPS nestlings, *n*=27; Control mothers/Control nestlings, *n*=32. Data are presented as least squares means±1 s.e.m. of untransformed data.

Maternal immunization increased the nestlings' wing-web swelling response to PHA (Maternal treatment: *F*_1,23.33_=5.399, *P*=0.029). Nestlings immunized previously with LPS showed a reduced wing-web swelling than did nestlings not previously immunized with LPS (Nestling treatment: *F*_1,88.15_=4.663, *P*=0.034; Fig. 3). There was a non-significant tendency for an interaction between maternal immunization and nestling immunization (*F*_1,88.15_=3.259, *P*=0.075), suggesting that maternal immunization may offset the reduced wing-web swelling experienced by nestlings previously challenged with LPS. There was no correlation between an individual's metabolic rate and subsequent response to PHA (RMR: β=−0.001, *F*_1, 32.27_=1.068, *P*=0.309).

### Maternal immunization increased fledging success but did not affect maternal return rates

**Fig. 4.**
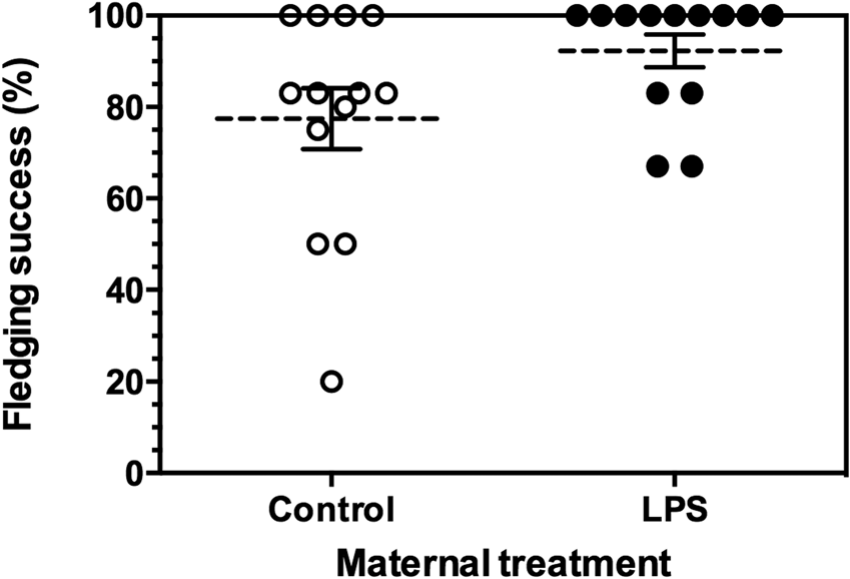
**Effect of maternal immunization** **with LPS on the percentage of offspring fledged from each nest.** Figure summarizes the fledging success of each individual nest, with mean (dashed line)±1 s.e (whiskers) indicated. Statistical analysis was performed on the survival of individual nestlings using a GLMM, with maternal identity as a random effect (to control for non-independence of brood mates), and with a quasi-binomial distribution (see text for details). Individual nestlings whose mothers had been immunized had greater probability of fledging (*P*<0.05). Sample sizes (number of nests): LPS mothers, *n*=13; Control mothers *n*=13. If the Control nest with 20% fledging success was omitted, the effect of maternal immunization was marginally significant (GLMM, *P*=0.06).

Fledging success (the number of hatchlings surviving until fledging) was significantly higher for chicks of LPS-immunized mothers than for chicks of control mothers (mean±s.e., LPS mothers: 92.3±3.6%, *n*=13 nests; Control mothers: 77.6±6.7%, *n*=13 nests; GLMM, t=−2.253, *P*=0.034, d.f.=24; Fig. 4). There was no effect of nestling immunization on fledgling success, nor any interaction between maternal and nestling immunization and fledging success (both *P*>0.40). If we excluded a single control nest with only 20% fledgling success, maternal immunization had a marginally significant effect on fledging success (GLMM, t=−1.969, *P*=0.061, d.f.=23). In total, 10 of 26 females (5 of 13 in each maternal treatment; 38%) returned to breed the following year, indicating no obvious negative effect of maternal immunization on female return rates.

## DISCUSSION

Maternal immunization had no long-term effect on growth rates, but affected the energy expenditure, immune function and fledging success of nestling tree swallows. When nestlings were challenged with LPS, all lost mass over the subsequent 24 h regardless of maternal treatment, suggesting that maternal immunization did not offset the short-term growth suppressive effects of mounting an acute phase response. Contrary to our prediction, there was no evidence that maternal immunization resulted in energetic savings for nestlings when individuals were subsequently challenged with the same antigen as their mothers. In fact, maternal immunization increased the energy expenditure of control nestlings. Nonetheless, nestlings from immunized mothers displayed a more robust inflammatory response to a novel antigen (PHA). This suggests either non-specific priming of the immune system or that LPS-immunized mothers invested more resources into their offspring during the pre-natal and/or nestling period. Either way, maternal immunization resulted in greater overall fledging success.

Immunization had no effect on the date a female initiated egg laying, her clutch size, or her probability of returning to breed the following season. This is consistent with previous immunization studies of birds, using similar dosages of LPS (e.g. Pied flycatchers, *Ficedula hypoleuca*, Grindstaff et al., 2006; House wrens, *Troglodytes aedon*, Bowers et al., 2012). The LPS antigen is non-replicating and, at the dosage given, elicits a brief (∼12 h) response (Koutsos and Klasing, 2001; Sköld-Chiriac et al., 2014). As such, maternal immunization was expected to have short-term effects with no long-term negative impact on a female's initial reproductive investment (Bowers et al., 2015).

Prior to immunization all adult females had detectable levels of LPS-specific antibodies. This was not necessarily surprising because LPS is a component of gram-negative bacteria that individuals have presumably been exposed to in the wild (Grindstaff et al., 2006). When females were recaptured during incubation there had been no increase in LPS-reactive antibodies. A lack of increase in response to a single LPS immunization was also reported for female house wrens (Bowers et al., 2012). Our inability to detect an increase may in part reflect the timing of our immunizations and sampling. We collected a blood sample approximately 17 days post-immunization, when females were incubating eggs. By this point a female's LPS-reactive antibody levels may simply have dropped to the background levels found in non-immunized females. In studies of captive individuals, where increased LPS-antibody levels post-injection have been detected, it was possible to provide pre-laying females with a booster injection to induce a secondary response (Grindstaff, 2008; Merrill and Grindstaff, 2014). However, a second immunization was not possible in our study. Although we detected an increase in total Ig levels between the initial capture and re-capture, this was unrelated to whether a female had been immunized with LPS or not, and simply suggests a general increase in total Ig levels between pre-laying and incubation.

To determine the level of matAb transferal, we collected blood samples from nestlings when they were 6 days post-hatch. At this age, neither total Ig nor LPS-reactive antibody levels differed between the offspring of LPS-immunized females and offspring of control females; a similar result to that reported for 5-day-old pied flycatcher nestlings (Grindstaff et al., 2006) and 11-day-old house wrens (Bowers et al., 2012). A lack of difference may reflect a relatively short biological half-life of maternal antibodies in these species (King et al., 2010). However, we detected a general increase in LPS-reactive and total Ig levels between days 6 and 14 post-hatch, presumably due to endogenous antibody production. Nonetheless, the rate at which nestlings produced antibodies did not differ with maternal treatment. Our results thus provide no evidence that maternally-derived LPS-reactive antibodies have a general, or antigen-specific, enhancive effect on the humoral immune system of tree swallow nestlings (within the time frame we studied). A lack of priming following maternal immunization with LPS was also reported in zebra finch nestlings (Merrill and Grindstaff, 2014). This could be a result of the fact that LPS-reactive antibodies in the female were mainly of the IgM rather than IgG type and that matAb transfer mainly includes the IgG type (Grindstaff et al., 2003), thus not resulting in any antigen-specific matAb-mediated priming of offspring immunity. However, note that in pied flycatchers, offspring of LPS-immunized females developed higher circulating total antibody levels, and a tendency toward more LPS-specific antibodies near fledging, than did offspring of control females, suggesting that LPS-induced matAb can have enhancive effects on offspring immunity (Grindstaff et al., 2006). Enhancive effects of T cell-dependent matAbs on the specific humoral response have been reported in other passerines (song sparrows, *Melospiza melodia*, Reid et al., 2006; house sparrows, *Passer domesticus*, Broggi et al., 2016), although suppressive effects can also occur (zebra finches, *Taeniopygia guttata*, Merrill and Grindstaff, 2014). The reason for the differing immune responses among passerines is unclear. However, the presence of circulating matAbs in offspring is widely recognized to inhibit the immune response to vaccination in humans and other animals (Siegrist, 2003; Niewiesk, 2014). Although the degree of inhibition generally increases with circulating matAb levels, even low, non-protective levels can still inhibit antibody production (Niewiesk, 2014). Increasing the dose of vaccine antigen in the neonate can offset the inhibition by matAbs (Siegrist, 2003). The differing responses across avian species and studies presumably reflects absolute levels and decay rates of maternal antibodies in the nestlings' circulation, T cell-dependent versus T cell-independent antigens and their doses, and the interactions between maternal antibodies and other maternally-derived compounds (Siegrist, 2003; Niewiesk, 2014; Broggi et al., 2016).

In nestlings, one of the proposed benefits of receiving specific matAbs is the reduction of an inflammatory response (and avoidance of growth suppression) if nestlings encounter the same antigen to which their mothers had been exposed (Grindstaff, 2008). When we challenged nestlings with LPS, most individuals suppressed growth for approximately 24 h post-injection. Contrary to our prediction, nestlings whose mothers had been immunized with LPS had the same degree of growth suppression as nestlings whose mothers had not been immunized. Broadly similar patterns have been reported in zebra finches (Grindstaff et al., 2012). In contrast, the presence of maternal antibodies partially offset growth suppression due to antigen exposure in precocial Japanese quail chicks (*Coturnix japonica*, Grindstaff, 2008). Our inability to detect an effect of maternal immunization on nestling growth rates is consistent with a lack of difference in levels of circulating LPS-antibody levels in 6-day-old nestlings of immunized and non-immunized mothers. As a result, the duration over which maternal antibodies might impact growth rates during an immune challenge is likely shorter in altricial than in precocial species (Grindstaff et al., 2012).

Across the nestling phase, we found that nestlings from LPS-immunized mothers grew at similar rates as nestlings from control mothers; a result also reported in zebra finches (Grindstaff et al., 2012) and house sparrows (Broggi et al., 2016). Although not differing statistically in growth rate, we found that nestlings of immunized mothers had longer wings (by about 1.2 mm) between days 9 and 14 post-hatch, than did nestlings of control mothers. Maternal immunization resulted in nestlings with longer wings in a previous study of cross-fostered tree swallows, a result presumably due to differences in egg constituents, e.g. matAbs or androgens (Lozano and Ydenberg, 2002). Recent work on house wrens found that nestlings of mothers exposed to LPS prior to egg laying had increased growth rates, but this was mediated by differences in maternal feeding rates, and was hypothesized to reflect terminal investment (Bowers et al., 2015). The difference we detected in wing length between days 9 and 14 was relatively small, and was no longer detectable when we isolated our analyses to nestlings of 14 days of age.

As an index of the energetic costs of mounting a response to LPS immunization, we measured the oxygen consumption of nestlings. We predicted that when nestlings were challenged with LPS, offspring of LPS-immunized mothers would have a lower metabolic rate than offspring of control mothers, because offspring of LPS-immunized mothers would avoid the energetic costs associated with mounting an innate immune response and instead rely on the LPS-specific matAbs to exterminate the LPS antigen. In contrast, nestlings challenged with LPS exhibited the same energy expenditure, irrespective of whether their mothers had been immunized or not. A lack of energetic savings is consistent with our inability to detect differences in LPS antibody levels of chicks from the different maternal treatments. Unexpectedly, maternal immunization resulted in an increase in the energy expenditure of control (unimmunized) nestlings. This suggests that in the absence of an LPS challenge, nestlings may pay an unavoidable energetic cost associated with maternal immunization. This is consistent with the idea that there is a fitness cost when environmental conditions experienced during one stage of development (and for which an individual has been ‘programmed’) are mismatched with conditions during subsequent stages (Monaghan, 2008; Sheriff et al., 2010). For example, recent work on zebra finches has explored the effect of matching and mismatching maternal and nestling antigen exposure on subsequent adult stress reactivity, finding that individuals mismatched across life stages had a heightened response to stress as adults (Merrill and Grindstaff, 2015). In our study, maternal LPS immunization may have ‘primed’ nestling tree swallows to cope with a subsequent LPS challenge, which, in the case of control nestlings, never emerged.

The source of the increase in energy expenditure in control nestlings is not known, but it may be associated with non-specific priming and reflect the cost of developing and/or maintaining a more robust immune system (van der Most et al., 2011). Although the activation costs of induced innate defenses are substantial, the developmental costs are thought to be relatively low (Lee, 2006). In contrast, the costs associated with development of induced adaptive defenses may be high, in part due to proliferation and diversification of lymphocyte (e.g. B cells, T cells, reviewed in Klasing and Leshchinsky, 1999; Lee, 2006). Because of increased energetic costs to nestlings, an acceleration of immunological development may only result in fitness benefits in environments with increased pathogen prevalence (e.g. Gasparini et al., 2001), and/or those in which there is sufficient food availability to offset increased parental foraging.

To measure oxygen consumption we isolated nestlings for approximately 4 h. We chose this time period to ensure the nestlings were post-absorptive, and that our metabolic measurements were not elevated by a heat increment of feeding (Burness et al., 2000). Nonetheless, we acknowledge that short-term food restriction can result in increased corticosterone levels (Saino et al., 2003), and that corticosterone has been linked with resting metabolic rates (Jimeno et al., 2017). If our respirometry procedure affected our estimates of metabolic rate, via glucocorticoid elevation, we presume nestlings from each treatment would have been impacted similarly (but see Merrill and Grindstaff, 2015).

Late in nestling development, we challenged all individuals with PHA, a compound inducing a complex inflammatory response, and involving aspects of both the innate and adaptive (cell-mediated) immune systems (Martin et al., 2006; Vinkler et al., 2010). Nestlings of LPS-immunized mothers had a more robust response to PHA (i.e. greater inflammation) than those of non-immunized mothers, suggestive of a general non-specific priming of immunity in offspring of LPS-immunized mothers. LPS is a potent antigen which can stimulate various components of the immune system, and has been hypothesized previously to result in general non-specific enhancement of humoral immunity in pre-laying birds (Hasselquist and Nilsson, 2009). In contrast, an enhanced PHA response was not detected in nestlings whose mothers had been challenged previously with Newcastle disease virus (Broggi et al., 2016) or sheep red blood cells (Lozano and Ydenberg, 2002), suggesting that priming effects are likely antigen-specific. In addition to antibodies, mothers can influence a nestling's response to PHA via adjustments of *in ovo* corticosterone, yolk (Bowers et al., 2015), and androgen levels (Hasselquist and Nilsson, 2012). However, because we did not sample eggs we are limited in our capacity to identify the proximate mechanism(s) underlying the response of nestling tree swallows. Nonetheless, numerous studies have shown that an individual's response to PHA correlates with post-fledging recruitment (Moreno et al., 2005; López-Rull et al., 2011; Bowers et al., 2014), suggesting potential fitness benefits.

A nestling's response to PHA was influenced by a non-significant trend (*P*=0.075) for an interaction between maternal immunization and nestling immunization. Nestlings immunized with LPS appeared to display an attenuated response to PHA if they were from control (non-immunized) mothers, but not if they were from LPS-immunized mothers. This suggests that mounting a response to one antigen (e.g. LPS) can have substantial negative carry-over effects for nestlings, but these effects can be ameliorated via maternal immunization. An additional explanation was suggested by an anonymous referee, who noted that control nestlings from control mothers had a similar response to PHA, as did nestlings of LPS-immunized mothers (both LPS and control nestlings). This suggests, that rather than there being a general non-specific priming of immunity in all offspring of LPS-immunized mothers, priming had the greatest effect in nestlings that had been challenged with LPS. If this were the case, an alternative/additional interpretation is that maternal immunization with LPS does not result in general non-specific priming, but instead counteracts the negative effects of an early-life LPS challenge in nestlings.

Nestlings from immunized mothers had higher fledging success than those from non-immunized mothers. Although we hypothesize that this effect was mediated by maternal immunization, our experimental design does not allow us to distinguish between prenatal effects (e.g. matAbs or other egg constituents), and differential maternal investment during chick rearing. For example, house wren females challenged with LPS pre-laying increased chick feeding rates, but had lower return rates the following year, consistent with a strategy of terminal reproductive investment (Bowers et al., 2015). However, in our study maternal immunization did not affect a female's clutch size, hatching success, mass of nestlings, nor a female's return rates the following year suggesting terminal investment is an unlikely explanation in our study system. We do not know the exact source of nestling mortality; however, it was not due to widespread depredation. Depredation rates are generally low at our study site, and when it has occurred, entire nests were destroyed (e.g. from racoons, *Procyon lotor*, Hogle and Burness, 2014). In the current study, nestlings that died were often recovered in the nest, and had frequently been the lightest member of the brood.

In conclusion, we hypothesized that maternal immunization would affect growth rates, energy expenditure, and immune function in nestling tree swallows. We did not detect a difference in either LPS-reactive antibodies or total Ig levels between offspring of immunized and non-immunized mothers, possibly reflecting a relatively short half-life of matAbs in altricial birds. We found no evidence that nestlings from LPS-immunized mothers could avoid the growth suppression that results from activation of an inflammatory response. Unexpectedly, we found that in the absence of an antigen challenge, nestlings of LPS-immunized mothers had higher resting metabolic rates than nestlings of non-immunized mothers. We attribute the increased RMR to the costs associated with general non-specific enhancement of immune function in nestlings from LPS-immunized mothers. Consistent with enhanced immune function, nestlings of LPS-immunized mothers had a more robust response to PHA, and higher fledging success. Our results suggest that maternal antigen exposure pre-laying can result in increased fitness for mothers through more robust immune responses, and higher fledging success of their offspring. However, this presumably can only occur in environments where food resources are sufficient to allow parents to fuel the increased energetic demands of their nestlings.

## MATERIALS AND METHODS

### Study area and species

All research was approved by the Trent University Animal Care committee, in accordance with the Canadian Council on Animal Care, with handling, banding and collection permits granted by Environment Canada (now, Environment and Climate Change Canada). This study was conducted in May-July 2009 and 2010, on a nest-box breeding population of tree swallows (*Tachycineta bicolor*) located at the Trent University Nature Area (44°21′N, 78°17′W) Peterborough, Ontario, Canada (Hogle and Burness, 2014). Nearly 100 nest boxes mounted on wooden stakes, 10-20 m apart, are in an open field area. Boxes have a base of 13 cm, 25-30 cm walls, a slanted roof and a 3.8 cm hole in the front. All nesting material is removed at the end of each breeding season. Females at this site lay clutches of typically five to seven eggs, with one egg laid each day during laying. Once a clutch is completed, females incubate the nest for approximately 14 days, and nestlings typically hatch synchronously. Nestlings typically fledge 18-22 days post-hatch (reviewed in Winkler et al., 2011).

### General field methods

Beginning in early May, nest boxes were visited at least once per week to record nest building activity. When a nest appeared to be at or near completion the female was trapped in the nest box, banded with an aluminum leg band (if necessary), weighed using a spring scale (±0.1 g), and had its wing chord measured using a wing rule with a stop at one end (±1.0 mm). Each female was then blood sampled (100 μl) from the brachial vein to measure background levels of antibodies, immunized (with LPS or saline, details below), and then released. Nest boxes were then checked daily to record date of clutch initiation and completion. Clutches were considered complete (incubation day 0) when no new egg was found on two consecutive days. On incubation day 7 (±1 day) the resident female at each nest box were re-captured, re-weighed, and a second blood sample (100 μl) was collected for measurement of LPS-reactive antibodies and total Ig levels. Only after-second-year females were used in this study; second-year females or males that were captured incidentally were banded and released.

Beginning on day 12 of incubation, eggs were checked daily to record hatching. Mean hatch date for the brood was the day when most of the nestlings in the nest hatched; if equal numbers hatched on two consecutive days, the first day was considered as the mean hatch day (Hatch=Day 0). We calculated hatching success as the percentage of eggs laid that hatched from a given clutch. Nestlings were banded on day 4 post-hatch. To monitor nestling growth, we measured the body mass and left wing length from elbow to wrist of nestlings on days 4, 6, 8, 9, 12, 14 and 15, using a Pesola spring scale (±0.1 g) and digital caliper (0.01 mm), respectively. We collected a blood sample (∼50 μl) from each nestling on day 6 (for maternally-derived antibodies), day 9 (as part of another study), and day 14 post-hatch (for residual maternal antibodies and nestling antibody production). Fledging success was determined after checking all nest boxes on day 20 post-hatch, and in the following days if necessary. Fledgling success was calculated as the percentage of hatchlings that successfully left the nest. Adult female return rates were estimated based on re-sightings the following years (2010 and 2011). It was not possible to estimate nestling recruitment, due to low return rates.

### Maternal and nestling immunization

When females were initially captured they were assigned to either the experimental-treatment or the control-treatment. Prior to immunization the skin surrounding the injection site was sterilized with ethanol. Experimental females then received an intra-abdominal injection of 20 μg Lipopolysaccharide (LPS, from *Escherichia coli*, Sigma-Aldrich L-2880) suspended in 100 μl sterile pyrogen-free phosphate buffered saline (PBS, Sigma-Aldrich P-5368). This concentration of approximately 1 mg kg^−1^ body mass has been shown previously to elicit mild sickness behavior and an acute phase response in passerines (Burness et al., 2010). Control females were handled identically, but were injected with 100 μl PBS.

When nestlings were 8 days old, one-half of the young in each nest received an intra-abdominal injection of 10 μg LPS suspended in 50 μl sterile pyrogen-free PBS (Sigma-Aldrich P-5368); the other half received 50 μl PBS. To minimize possible effects of laying order, we followed Grindstaff et al. (2006) and weighed all nestlings within a brood prior to immunization. Beginning with the heaviest nestling, we assigned treatment based on mass; alternating treatments in sequence from the heaviest to the lightest nestling, until all members of the brood had been immunized. The heaviest nestling in the next brood was then allocated to the alternate treatment. Immunization of most nestlings in a brood occurred in the field, however, individuals to be used in metabolic rate trials (details below) were immunized in the lab.

### Nestling metabolic rate

To test whether maternal immunization influenced the energetic cost of mounting an immune response in nestlings, we measured the oxygen consumption rate of a subset of nestlings. On day 8 post-hatch, two nestlings from each nest, matched for size, were removed from their nest box and transported in a cloth bag to the respirometry laboratory (<30 min). Each nestling was weighed (±0.01 g), injected with LPS or saline (details above), and placed in a 700 ml Plexiglas respirometry chamber (Model G114, QUBIT Systems, Kingston, Canada). The metabolic chamber containing each bird was then placed in a darkened, temperature-controlled incubator (Thermo Low Temperature Incubator, Model 815, Thermo Fisher Scientific), set at 32.5°C, which is within the thermo-neutral zone of adult tree swallows (Williams, 1988). Nestlings then had their oxygen consumption rate measured over the subsequent 4.0-4.5 h using an open flow, push-through respirometry system, following, Burness et al. (2010). Briefly, outdoor air was scrubbed of water and CO_2_ and directed either into one of the metabolic chambers containing a bird or into a piece of Bev-A-Line tubing to measure baseline. Flow rates were ∼200 ml min^−1^. Air exiting the chamber was scrubbed of water vapor and CO_2_, and then directed through the oxygen analyzer (FC-10a O_2_ Analyzer, Sable Systems, Las Vegas, USA). At the end of the trial, each individual was removed from its metabolic chamber and transported back to its nest box where it was placed back in the nest. The respirometry system could measure up to three nestlings simultaneously. Although typically only two nestlings were measured at a time, on busy days a third nestling from a different nest was included in the trial. This resulted in the two nestlings from this extra nest being measured during different trials (although in the same day). During each recording session we first measured 5 min of baseline, 15 min of air from a chamber containing a bird, followed with another 5-min baseline measurement. Measurements alternated among the chambers over a trial, such that each nestling's oxygen consumption was measured for at least 60 min in total. Resting oxygen consumption rate was determined from the lowest stable 5-min period of continuous oxygen consumption for each nestling, using LabAnalyst X (Warthog Systems, available at: http://warthog.ucr.edu/), and calculated using Eqn 10.1 from Lighton (2008). We could not be certain nestlings were post-absorptive, but we assumed any heat increment of feeding would be similar across maternal treatments.

### Nestling wing-web swelling response

Following blood sampling of nestlings on day 14, each nestling underwent a PHA skin test. PHA is derived from kidney beans and it induces a complex inflammatory response, likely involving aspects of both the innate and adaptive immune systems (Vinkler et al., 2010). The thickness of each nestling's left (non-blood sampled) wing web was measured (pre-injection thickness) using a digital micrometer (227-211, Mitutoyo, Japan ±0.001 mm) and then subcutaneously injected with 0.1 mg of PHA (PHA-P, Sigma-Aldrich L-8754) in 20 μl of sterile saline (Sigma-Aldrich P-5368), as per Smits et al. (1999). The wing web thickness was re-measured on day 15, 24 h after the injection. The average of triplicate repeated measures was typically used, although in some instances, four measurements were taken. The skin swelling response was calculated as the difference between the average post- and pre-injection thicknesses.

### Blood sampling and storage

All blood samples were collected by puncturing the brachial vein, and collecting the blood into heparinized microcapillary tubes. The tubes were sealed with Critoseal, labeled, and placed in a chilled cooler until they were centrifuged up to 4 h later (Damon/IEC MB Micro Hematocrit centrifuge, Thermo Fisher Scientific). The plasma was removed from the capillary tubes using a Hamilton syringe, transferred into labeled microcentrifuge tubes, and stored at −80°C until analysis.

### LPS-reactive antibodies and total IgG

As a measure of humoral immunity, we quantified LPS-reactive antibodies and total Ig using an enzyme-linked immunosorbent assay (ELISA). Plasma samples from adults and nestlings were analyzed for LPS-reactive antibodies following Sköld-Chiriac et al. (2014) and total Ig following Grindstaff et al. (2006) and Sköld-Chiriac et al. (2014). Briefly, for analysis of LPS-reactive antibodies, 96-well ELISA plates were coated with 100 µl LPS diluted to 5 µg ml^−1^ in carbonate buffer (0.15 M, pH 9.6). For analysis of total Ig, plates were coated with 100 µl of anti-chicken IgG (donkey anti-chicken IgG, 67-645, ICN Biomedicals), diluted in carbonate buffer to 6 µg ml^−1^. Plates were incubated at 4°C overnight, and the following day were blocked for at least 2 h at room temperature using 3% milk powder, diluted in 0.01 M phosphate buffered saline (pH 7.2) and Tween 20. To each well, 100 µl of plasma diluted in diluent (1% powdered milk in PBS-Tween 20) was then added, in duplicate. Adult and nestling plasma samples for analyses of LPS-specific Ab were diluted 1:200, while samples for assays of total Ig levels were diluted 1:400. All plates also included two blanks containing only diluent, and a series of diluted plasma standards. These standards were created from pooled plasma samples, and spanned the range of antibody concentrations in the nestling and adult samples. All plates were again incubated overnight at 4°C. On the third day, 100 µl of rabbit-anti-red-winged blackbird IgG (diluted 1:1000 in diluent) was added to each well. This secondary antibody has been used successfully to detect antibodies in a previous study of tree swallows (Hasselquist et al., 2001). Plates were then incubated at 37°C for 1 h. Following incubation, 100 µl of peroxidase-labelled goat-anti-rabbit serum antibody (Sigma-Aldrich A6154) diluted 1:2000 in diluent was then added to each well, and plates were incubated at 37°C for 30 min. Between each step, plates were washed in an ELISA plate washer (ELx50, BioTek). Finally, 100 µl of peroxidase substrate (2,2-azino-bis-3-ethylbenzothiazoline-6-sulfonic acid, ABTS; Sigma Aldrich A1888) and peroxidase diluted in citrate buffer (pH 4.0) were added to each well, and plates were read using a kinetic ELISA reader (BioTek EL 808) at 405 nm, every 30 s for 14 min. Values are reported as the slope of the substrate conversion over time; a steeper slope indicating more antigen-specific antibodies in the plasma sample. Antibody titers were calculated as the mean of the duplicate samples, subtracted from the mean of the duplicate blanks. To account for variation among plates, we used the differences between the diluted plasma standards curves that were run on each plate.

### Statistical analysis

All data are available from the Dryad data repository at https://doi.org/10.5061/dryad.6bn74m7. All statistical analyses used JMP v10 (SAS Institute Inc., Carey, USA; 1989-2012), or R (R Core Team, 2013), and statistical significance was claimed at *P*<0.05. During field and laboratory work we were generally blinded to the treatments groups, although not during subsequent statistical analyses. Sample sizes were based on the number of females that could be successfully captured pre-egg laying. Nests were included only if females had laid their first egg at least 5 days post-immunization, because antibodies are known to be produced by this time (Sköld-Chiriac et al., 2014). Data were transformed as necessary to reduce heteroscedacity and/or improve the normality of residuals. Maternal LPS-reactive antibody levels were Log10 transformed, as were nestling LPS-reactive antibody and total Ig levels. Wing-web swelling data were arcsine-square root transformed. All other data remained untransformed.

We analyzed the effects of maternal immunization on clutch initiation date and clutch size using general linear models, with maternal treatment and year as main effects. To analyze the effect of maternal immunization on nestlings we used general linear mixed models (GLMMs) with maternal ID as a random effect, and maternal treatment, nestling treatment and their interaction as fixed effects. In analyses with repeated measurements of nestlings, we also included nestling ID as a random effect. We initially included hatching date, year of study, brood size, and all two-way interactions. To obtain the most parsimonious models, we removed non-significant interaction terms followed by non-significant main effects, and re-ran the models. Because we were particularly interested in the main effects of maternal treatment and nestling treatment, as well as their interaction, these terms were always retained in the model. To account for possible differences among females in the time available to generate antibodies prior to egg laying, once the most parsimonious models for nestlings were identified, we checked for an effect of the number of days elapsed between maternal immunization and the laying of a female's first egg; if this term was not significant, it was then eliminated. When reporting nestling antibody levels, we provide the statistical output for the number of days elapsed (despite being a non-significant covariate); for all other analyses we reported the output for this covariate only when significant. In analyses of oxygen consumption rate, we also included nestling body mass as a covariate. Criteria for identifying possible outliers were decided prior to data analysis, and involved scrutinizing any individual with a Studentized residual<−3.0 or>+3.0 (following Burness et al., 2013; Hogle and Burness, 2014). One nestling had an unusually high oxygen consumption rate, suggesting it was not at rest during the metabolism trial (Studentized residual=+3.26). We excluded this individual from further analysis of oxygen consumption. Inclusion of this individual increased the *P*-value of the maternal treatment×nestling treatment interaction from *P*=0.03 to *P*>0.15.

To statistically compare hatching and fledgling success across treatments we constructed a GLMM using the glmmPQL function from the ‘MASS’ package in R (R Core Team, 2013). We used a quasi-binomial distribution due to under-dispersion of the data. We included maternal ID as a random effect, and maternal treatment as a fixed effect. In analyses of fledging success, we also included nestling treatment and the interaction between maternal and nestling treatment as main effects. Inclusion of year did not add to the predictive ability and was subsequently excluded. In the results, we report hatching and fledging success as percentages, but the corresponding statistical output was from the GLMM.
